# Shoulder ligamentoplasty, arthroscopic Latarjet, dynamic anterior stabilization, and arthroscopic Trillat for the treatment of shoulder instability: a systematic review of original studies on surgical techniques

**DOI:** 10.1186/s13018-025-06422-7

**Published:** 2025-11-11

**Authors:** Carlos Galindo-Rubín, Yehinson Barajas Ramón, Fernando Maniega-Legarda, Álvaro Velarde-Sotres

**Affiliations:** 1 Traumatología y Ortopedia, Hospital Universitario Marqués de Valdecilla, Santander, Spain; 2 Facultad de Ciencias de la Salud, Universidad Europea del Atlántico, Santander, Spain; 3 Departamento de Salud, Universidad Internacional Iberoamericana, Campeche, Mexico; 4 Faculdade de Ciências de Saúde, Universidade Internacional do Cuanza Bairro Kaluanda, Cuito, Angola

**Keywords:** Ligamentoplasty, Arthroscopic Latarjet, Dynamic anterior stabilization, Trillat procedure, Sports injuries, Shoulder instability

## Abstract

**Background:**

Anterior shoulder instability is a common condition, especially among young and active individuals, often associated with both osseous and soft tissue injuries. Recent innovations have introduced various surgical options for managing critical and subcritical instability. Therefore, the primary objective of this systematic review was to collect, synthesize, and integrate international research published across multiple scientific databases on shoulder ligamentoplasty, arthroscopic Latarjet, dynamic anterior stabilization (DAS), and arthroscopic Trillat techniques used in the treatment of shoulder instability.

**Method:**

A structured search was conducted following the Preferred Reporting Items for Systematic Reviews and Meta-Analyses (PRISMA) guidelines and the PICOS model, up to January 30, 2025, in the MEDLINE/PubMed, Web of Science (WOS), ScienceDirect, Cochrane Library, SciELO, EMBASE, SPORTDiscus, and Scopus databases. The risk of bias was evaluated, and the PEDro scale was used to assess methodological quality.

**Results:**

The initial search yielded a total of 964 articles. After applying the inclusion and exclusion criteria, the final sample consisted of 25 articles. These studies demonstrated a high standard of methodological quality. The review summarized the effects of ligamentoplasty, arthroscopic Latarjet, dynamic anterior stabilization, and arthroscopic Trillat techniques in treating shoulder instability, detailing the sample population, immobilization period, frequency of instability episodes—including recurrent dislocations and subluxations—surgical methods, study designs, assessed variables, main findings, and reported outcomes.

**Conclusions:**

Arthroscopic ligamentoplasty is advantageous in preserving the patient’s native anatomy, maintaining joint integrity, and allowing for alternative interventions in case of failure. The arthroscopic Trillat technique offers a minimally invasive solution for anterior instability without significant bone loss. The DAS technique utilizes the biceps tendon to provide dynamic stabilization, aiming to generate a sling effect over the subscapularis muscle. The Latarjet procedure remains the gold standard for managing anterior glenoid bone loss greater than 20%. Each surgical option for anterior shoulder instability carries specific implications, and treatment decisions should be tailored based on bone loss severity, capsuloligamentous quality, and the patient’s functional needs.

## Introduction

Recurrent anterior shoulder instability involves repeated glenohumeral dislocations that often require reduction maneuvers. Its estimated incidence is 1.7%, with traumatic etiology accounting for approximately 90% of cases [1, 2]. This condition significantly impairs quality of life, leading to chronic pain, functional limitations, and restrictions in daily activities [3, 4]. It is frequently associated with structural osseous and capsuloligamentous abnormalities that compromise joint stability [4].

Key osseous defects include Hill-Sachs lesions and anteroinferior glenoid bone loss [4]. The most common capsuloligamentous injury is the Bankart lesion, while HAGL lesions may also occur but are often underdiagnosed [4]. Proper assessment of risk factors such as young age, history of dislocations, ligamentous laxity, muscular and neurological conditions, and high-demand activity is essential for treatment selection [5].

Due to its wide mobility and limited intrinsic stability, the shoulder is prone to recurrent dislocation after trauma [4]. Most primary dislocations show osseous involvement: 86% with glenoid defects, 94% with Hill-Sachs lesions, and 81% with both [6]. These injuries frequently coexist with capsulolabral damage of variable extent [7].

Arthroscopic Bankart repair is considered the standard approach in patients without significant bone loss and with stable Hill-Sachs lesions [8]. However, the threshold of bone loss requiring augmentation remains debated, and no universal classification exists for stratifying soft tissue injuries [7]. Glenoid defects >20% are regarded as critical, but even losses of 13.5% may compromise outcomes if untreated [9]. Soft tissue quantity and quality also determine repair success [10]. In addition, the on-track/off-track concept, formalized by Di Giacomo and colleagues [11], refines risk stratification by comparing the effective glenoid track (the area of glenoid contact that narrows with anterior bone loss) with the Hill–Sachs interval. A lesion is on-track when it remains covered by the glenoid track during functional motion (less likely to engage and often suitable for soft-tissue repair alone), and off-track when it extends beyond this track (prone to engagement/recurrence).

In cases of minor osseous or ligamentous deficits (including combined lesions), Bankart repair, with or without Remplissage, is preferred and achieves favorable outcomes, with similar results using bioabsorbable or metallic anchors [12]. When bone loss exceeds 20%, or in cases of recurrent instability or major tissue insufficiency, reconstruction techniques such as Latarjet or bone grafting are recommended despite higher complication rates [9]. The main therapeutic dilemma is moderate bone loss, where Bankart with Remplissage and Latarjet yield similar recurrence, but Latarjet shows higher complication (up to 30%) and reoperation rates (up to 7%) [13–15].

Di Giacomo et al. [16] proposed an algorithm for treating anterior instability based on the extent of glenoid bone loss. A glenoid bone loss of less than 15% favors soft tissue repair, whereas losses between 17 and 25%, the so-called “gray zone”, require individualized surgical planning. Sedentary individuals or non-contact athletes may benefit from isolated arthroscopic repair, while contact athletes achieve better outcomes with glenoid reconstruction. Shaha et al. [9] introduced the concept of subcritical bone loss, showing that glenoid bone loss exceeding 13.5% results in poor functional outcomes with Bankart-only repair, even in the absence of recurrent dislocations. This finding suggests that, in active patients, soft tissue repair alone may be insufficient for long-term success.

Over the past two decades, shoulder instability treatment has evolved from open surgery to advanced arthroscopic techniques. The development of arthroscopic Latarjet, DAS, arthroscopic ligamentoplasty, and Trillat procedures has expanded treatment options, improving joint stability, reducing complications, and optimizing recovery [17, 18]. These techniques bridge the gap between open surgery, which offers greater stability but carries a higher risk of complications [19–21], and conventional arthroscopic repairs, which are less invasive but may have higher recurrence rates in subcritical bone loss cases. They offer personalized, hybrid solutions that optimize clinical outcomes while minimizing surgical risks [17, 18].

Among these, arthroscopic Latarjet has been validated [22] as a reliable technique, restoring glenoid bone loss and enhancing dynamic stability through a combined bony block and sling effect, while reducing morbidity compared to open procedures. Arthroscopic ligamentoplasty [23] reinforces anterior capsular stability using a synthetic graft or allograft anchored at the anteroinferior glenoid and passed through or over the subscapularis. The arthroscopic Trillat procedure [24, 25] repositions the coracoid to optimize tension in the conjoint tendon, and DAS [26] transfers the biceps tendon to the anterior glenoid, mimicking the sling effect through a less invasive approach.

Currently, no Level 1 A studies directly compare these surgical techniques for treating shoulder instability. However, existing evidence suggests that their outcomes are comparable, supporting their role as viable alternatives in managing severe instability [26, 27, 29].

Therefore, the main aim of this systematic review was to collect, synthesize, and integrate international research published across various scientific databases on shoulder ligamentoplasty, arthroscopic Latarjet, dynamic anterior stabilization, and arthroscopic Trillat techniques for the treatment of shoulder instability. This way, this review determines the current state of the knowledge about this topic and allows a better understanding of the existing problems, making easier the development of future lines of research.

## Methods

### **Searching strategies and sources of information**

This article presents a systematic review focused on surgical techniques for the treatment of recurrent anterior shoulder instability. The review was conducted following the Preferred Reporting Items for Systematic Reviews and Meta-Analyses (PRISMA^®^) guidelines [27], ensuring methodological integrity. The review was registered in PROSPERO (ID = CRD420251014582). Methodological issues were addressed using the guidance provided by the Cochrane Handbook for Systematic Reviews of Interventions [30].

**Fig. 1:**
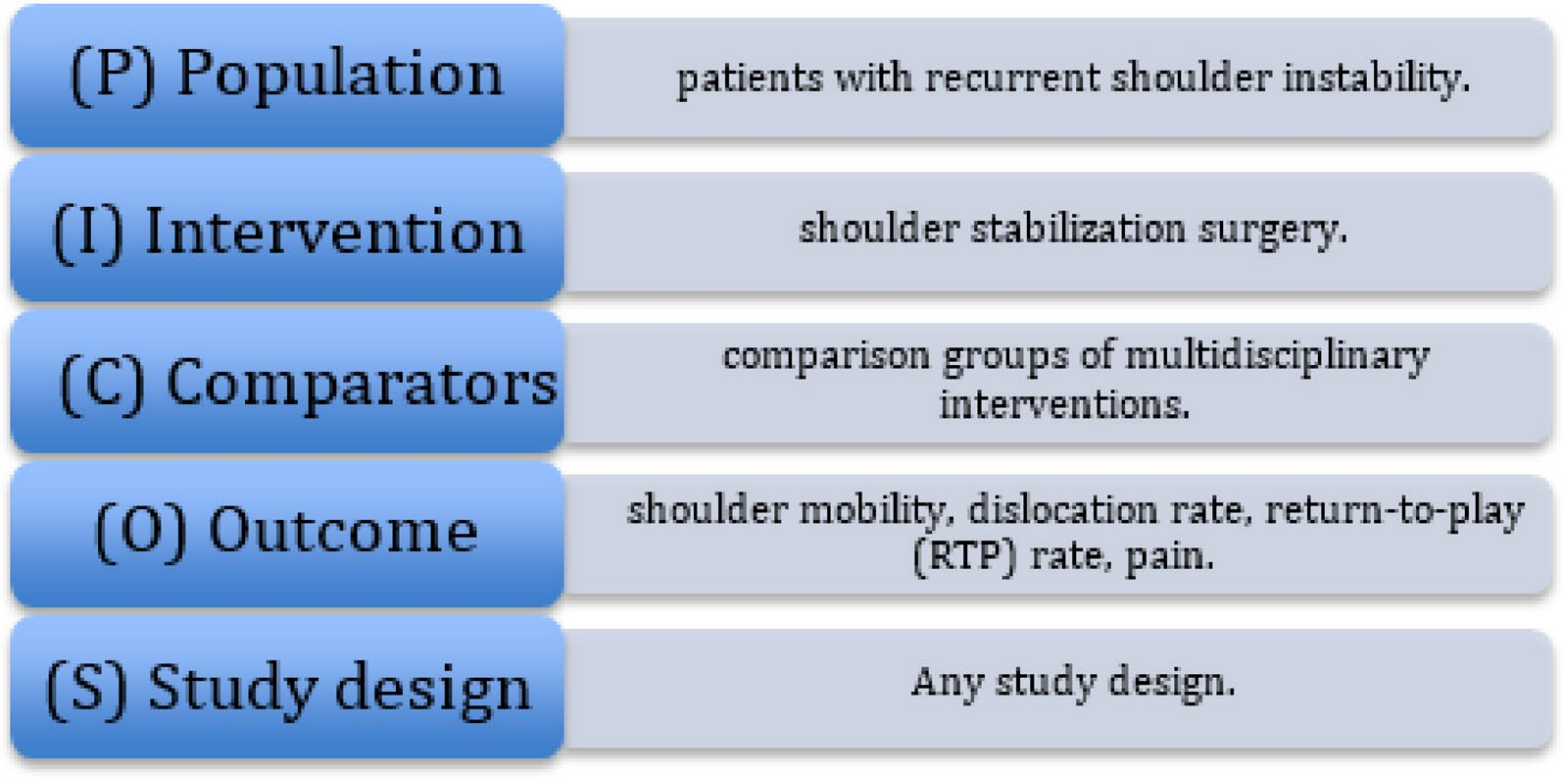
PICOS Model

The PICOS^®^ model was used to define the inclusion criteria [31]: P (Population): “patients with recurrent shoulder instability,” I (Intervention): “shoulder stabilization surgery,” C (Comparators): “comparison groups of multidisciplinary interventions,” O (Outcome): “shoulder mobility, dislocation rate, return-to-play (RTP) rate, pain,” and S (Study design): “any study design” (Fig. 1).

A structured search was conducted in MEDLINE/PubMed, Web of Science (WOS), ScienceDirect, Cochrane Library, SciELO, EMBASE, SPORTDiscus, and Scopus. The search was completed on January 30, 2025. Search terms included a combination of Medical Subject Headings (MeSH) and free-text keywords related to concepts such as shoulder instability, surgical techniques, and arthroscopic procedures. Specifically, the following search equation was used: [“*shoulder instability*” (MeSH Terms) OR “*shoulder dislocation*” (All Fields)] AND [“*Latarjet outcomes*”] AND [“*Dynamic Anterior Stabilization Shoulder*” (MeSH Terms)] AND [“*Trillat Shoulder*” (MeSH Terms)] AND [“*Shoulder Ligamentoplasty*” (MeSH Terms)]. This searches equation retrieved all relevant articles in the field. Additionally, the reference sections of the included articles were examined using the “snowballing method” [29], which involves reviewing the citations within retrieved studies. All titles and abstracts from the search were screened to identify duplicates and any potentially missing studies (C.G.-R. and A.V.-S). Titles and abstracts were selected for full-text review. The search for published studies was conducted independently by two different authors (C.G.-R. and A.V.-S), and any disagreements were resolved through discussion between them.

### Inclusion and exclusion criteria

Studies reporting effectiveness outcomes in terms of diagnostic accuracy or performance related to the surgical management of shoulder instability were selected. The systematic review included original studies on surgical techniques for treating severe shoulder instability. Systematic reviews, meta-analyses, conference abstracts, opinion pieces, and posters were excluded. Only studies with a minimum sample size of 10 participants were included. For effectiveness studies, only those that employed at least one arthroscopic shoulder stabilization technique were considered. The surgical techniques examined for comparison included arthroscopic Latarjet, arthroscopic Trillat, Dynamic Anterior Stabilization, and shoulder ligamentoplasty.

For the articles retrieved during the search, the following inclusion criteria were applied to the final selection: (I) studies published in peer-reviewed journals with full-text availability; (II) articles analyzing the effects of stabilization surgeries in patients with shoulder instability; (III) original research articles published in peer-reviewed journals with an impact factor; (IV) studies involving patients treated with arthroscopic Latarjet, arthroscopic Trillat, Dynamic Anterior Stabilization, or shoulder ligamentoplasty; (V) study populations consisting of patients who underwent arthroscopic techniques for the treatment of recurrent shoulder instability; (VI) studies that included preoperative and/or postoperative clinical evaluations; (VII) studies published in English and/or Spanish. The following exclusion criteria were applied to experimental research protocols: (I) lack of reliable measurements; (II) studies with fewer than 10 participants; (III) use of open surgical techniques; (IV) fixation methods involving metal buttons or staples; (V) abstracts, non-peer-reviewed articles, and book chapters.

### Study selection

Titles and abstracts of publications identified through the search strategy were reviewed for full-text selection and cross-checked to eliminate duplicates. All studies assessed for eligibility and classified as relevant were retrieved, and their full texts were peer-reviewed by two authors (C.G.-R. and A.V.-S). Additionally, the reference sections of all relevant articles were examined using the snowballing strategy [30]. Based on the information provided in the full-text articles, inclusion and exclusion criteria were applied to select eligible studies for inclusion in this systematic review. Any disagreements were resolved through discussion between the two authors (C.G.-R. and A.V.-S).

### Data extraction

Once the inclusion and exclusion criteria were applied to each study, the following data were extracted: study source (author(s) and year of publication); sample population, including number of participants; weeks of immobilization; recurrence or dislocation rate; surgical technique used; type of study design; variables analyzed; results and conclusions; and intervention effects.

For each study, information was carefully collected from all eligible publications. Mean (±), standard deviation (SD), and sample size data were extracted from the tables of all included studies. Disagreements were subsequently resolved through discussion until consensus was reached.

### Quality assessment and risk of bias

Methodological quality and risk of bias were assessed independently by two authors (C.G.-R. and A.V.-S), with any disagreements resolved by a third reviewer (J.C.-G), in accordance with the Cochrane Collaboration Guidelines [32].

The Cochrane Risk of Bias tool included the following domains: [1] selection bias (random sequence generation, allocation concealment) [2], performance bias (blinding of participants and personnel) [3], detection bias (blinding of outcome assessment) [4], attrition bias (incomplete outcome data) [5], information bias (selective reporting), and [6] other sources of bias.

For each investigation, the criteria were shown as “low” if the criteria were met for low-risk bias (unlikely to seriously alter the results) or “high” if the criteria were for high-risk bias (seriously undermining the reliability of the results). If the risk of bias was unknown, it was considered “unclear” (it casts doubt on the results).

The systematic review followed the principles of the PRISMA^®^ statement [30], a checklist designed to ensure transparency in systematic reviews and to improve their scientific credibility. PRISMA^®^ includes 27 items and a flow chart with four stages, which includes items considered essential for the transparent communication of a systematic analysis.

The Physiotherapy Evidence Database (PEDro) scale was also used to assess the methodological quality of all selected studies. This scale is a commonly used tool in literature reviews and is designed to evaluate the quality of clinical trial designs (Table 1). It is based on a list developed by Verhagen [35] using the Delphi technique [36].

**Table 1:** “Physiotherapy evidence database (PEDro) scale to analyze the methodological quality of the studies”

PEDro scale			
1	The selection criteria were specified	Yes	No
2	The subjects were randomly assigned to the groups	Yes	No
3	The allocations were undisclosed	Yes	No
4	The groups were similar at baseline in relation to the indicators of prognosis	Yes	No
5	All subjects were blinded	Yes	No
6	All the sports scientists providing therapy were blinded	Yes	No
7	All assessors evaluating at least one of key results were blinded	Yes	No
8	All the measures of at least one of the key results were obtained from more than 85% of the subjects initially assigned to the groups	Yes	No
9	The results of all the subjects receiving treatment or assigned to the control group were given, or when not possible, the data for at least one key result were analyzed “in order to treat”	Yes	No
10	The results of statistical comparisons among groups were reported for at least one key result	Yes	No
11	The study provides specific and variability measures for at least one key result	Yes	No

The PEDro scale has a total of 11 items. Item 1 refers to the external validity of the study, while items 2–9 refer to internal validity. Items 10 and 11 indicate whether the statistical information provided by the authors allows for accurate interpretation of the results. All items on the list are dichotomized as “yes”, “no”, or “not reported”. Each item marked “yes” receives one point, while items marked “no” or “not reported” receive no points. The first item of the PEDro scale was not considered in this review, as it relates to the evaluation of external validity. Therefore, only items 2 through 11 were used to assess methodological quality. As a result, the maximum possible score for an article was 10 points, and the minimum possible score was 0.

The evaluation of heterogeneity was another aspect considered in the review. Clinical heterogeneity refers to differences among types of patients, treatments, and outcomes. Methodological heterogeneity refers to variability in study designs and bias control.

## Results

### Main search

The database search identified 963 publications. Of the 963 articles retrieved, 16 were excluded given that they were duplicates. A digital screening of sources generated 807 relevant studies, which were included for review. After a detailed review of titles, abstracts, and full texts, 64 publications met the inclusion criteria. Subsequently, 40 articles were excluded for not addressing the surgical techniques under review or for being editorials, surgical technique descriptions, citations, reviews, posters, commentaries, or comparative studies. Studies involving epileptic patients were also excluded.

**Fig. 2:**
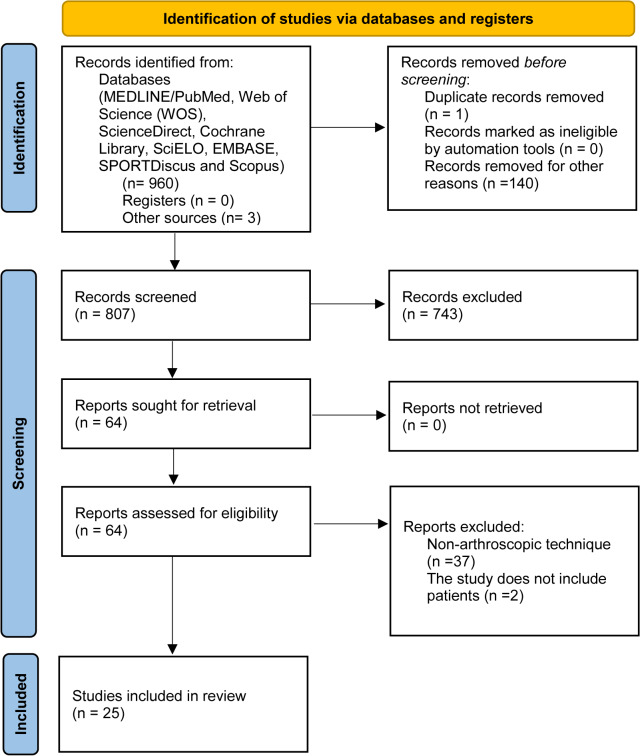
Flow diagram of the study selection

From the final selection, 25 studies were included in the systematic review. Seven articles contained significant data on the arthroscopic Trillat technique [37–43]. Four articles reported significant findings related to the Dynamic Anterior Stabilization technique [44–47]. Three articles presented relevant data on anterior shoulder ligamentoplasty [23, 48, 50]. Eleven articles provided significant information on the arthroscopic Latarjet technique [51–61] (Fig. 2).

### Study characteristics

The source of each study (author and year of publication), immobilization time, number of episodes of instability including recurrent dislocations and subluxations, surgical technique, type of study design, study variables, main findings, and the effects of the intervention are summarized in Table 2. A total of 25 original articles were included with significant data related to the arthroscopic techniques analyzed, specifically Trillat, DAS, arthroscopic Latarjet, and shoulder ligamentoplasty procedures.

**Table 2:** Methodology and results of the interventions

Study	N	Time	Eps	Surgical technique	Design	Variables	Main Findings	Effects
Labattut et al. [37]	18	4 wks	1	Arthroscopic Trillat 1 screw	Retrospective descriptive (at least 1 year follow-up)	Walch-Duplay score Rowe score External rotation loss Patient satisfaction Positive lift-off test Intra-operative complications	Satisfactory short- and mid-term stability, simple procedure, short operative time, no specific complications	→
Gonnachon et al. [38]	58	4 wks	no info	Arthroscopic Trillat	Single center, retrospective study	Morphological parameters were measured on all the rotator cuff muscles: cross sectional area (CSA), thickness and fatty infiltration using the mean muscle attenuation (MMA) measurement. Isokinetic tests were done 1 year post-surgery	Minor subscapularis atrophy at 6 months, no strength deficit at 1 year, likely screw-related	↑
Chauvet et al. [39]	52	3 wks	2 (3.8%)	Arthroscopic Trillat	retrospective	Constant Rowe Walch-Duplay subjective shouldervalue shoulder range of motion xRay	Good outcomes for chronic anterior instability; not recommended for > 20% glenoid loss	↑
Kazum et al. [40]	19	??	0 (o%)	Arthroscopic Trillat	retrospective review	Constant-Murley Walch-Duplay ROWE Subjective Shoulder Value (SSV) Visual Analogue Scale (VAS). Post-operatively, healing of the coracoid osteoclatsy was evaluated by CT scan	Effective for recurrent instability and apprehension in anterior/inferior hyperlaxity	
Boileau et al. [41]	30	4 wks	3 (10%)	Arthroscopic Trillat technique	retrospective evaluation of patients	x-rays computed tomography scans Subjective Shoulder Value visual analog scale Walch Constant Rowe	Effective for young athletes with hyperlaxity and no major bone loss; enables return to sports	↑
Boileau et al. [42]	21	4 wks	1 (4%)	Arthroscopic Trillat technique	Twenty-one consecutive patients retrospectively reviewed	x-rays computed tomography scan Subjective Shoulder Value visual analog scale Walch Constant Rowe	Durable option for recurrent dislocations in older patients with chronic MIRCTs and preserved motion	↑
Moore et al. [43]	74	3 wks	3 (4.1%)	Arthroscopic Trillat technique	Multicenter retrospective study	Dislocation recurrence. Subluxation recurrence Functional outcomes Time and level of return to sport Bony fusion complications. Constant Rowe Walch Duplay Shoulder Subjective Value	Highly effective for athletes with chronic instability; enables rapid return to sport	↑
De Campos et al. [44]	15	3 wks	1 (6.7%)	Arthroscopic DAS	unicentric single-arm prospective study	Western Ontario Shoulder Instability Index Rowe score range of motion strength ability to return to play at same level lack of recurrence of instability successful LHB healing lack of complications	DAS improves function, ensures LHB healing, and is safe for AGI with 20% GBL	↑
Collin et al. [45]	22	1.5 wk (10d)	3 (13.6%)	Arthroscopic DAS	A retrospective evaluation	Rowe score range of motion (ROM) recurrence	DAS supports Bankart repair in subcritical bone loss; preserves ROM, no Popeye deformity	↑
Wu et al. [46]	63	6 wks	0 (o%)	Arthroscopic DAS	retrospective cohort study	patient-reported outcomes range of motion return to sports (RTS) Postoperative recurrent instability complications	DAS-LHB and DAS-CT show similar recurrence, complications, STR, and function	→
De Campos et al. [47]	3	3 wks	0 (0%)	Arthroscopic DAS	single-arm prospective study	FF Abd E R IR WOSI RoweScore Shoulder Abduction Strength (kg)	Significant improvement in WOSI and Rowe scores (above MCID) MRI: Successful LHB tendon healing at glenoid	↑
Cuellar et al. [48]	52	1 wk	3 (5.8%)	Arthroscopic ligamentoplasty	retrospective descriptive study	Constant-Murley Score, subjective outcomes, radiographic control, ROM, apprehension signs, relocation tests, and shoulder laxity (anterior, posterior, inferior 'sulcus' tests)	59.6% excellent outcomes with no pain, full mobility and return to sports; 90% patient satisfaction	→
Sanchez et al. [49]	110	1 wk	2 (1.8%)	Arthroscopic ligamentoplasty	retrospective descriptive study	Constant score	Safe arthroscopic technique with good results	↑
Sánchez et al.[50]	168	1 wk	6 (3.57%)	Arthroscopic ligamentoplasty	multicenter retrospective study	Constant score degree of subjective satisfaction Stability mobility pain RTP reoperation complications	Good objective and subjective outcomes. This technique expands stabilizing surgical options	↑
Descamps et al. [51]	68	4 wks	4 (6%)	Arthroscopic Latarjet Procedure with Button Fixation	Single-Center Retrospective Study	Radiography (RX) Computed Tomography (CT) Rowe Score Age Sex Hyperlaxity ISIS Score Sports Bilateral Instability Previous Failed Soft Tissue Surgery Smoking Status Glenoid Bone Loss, Hill-Sachs Lesion	Safe, durable for recurrent instability; high RTS, minimal OA; suture button lowers complications vs screws	→
Dumont et al. [52]	64	1 wk	1 (1.5%)	**Arthroscopic** Latarjet procedure	Clinical retrospective study	Dislocations, subluxations, reoperations, WOSI score, and 15% complication rate in 64 patients	Low recurrence; better than Bankart, comparable to open Latarjet; reliable but technically demanding	↑
Boileau et al. [53]	47	4 wks	0 (0%)	Arthroscopic Latarjet procedure	Forty-seven consecutive patients Level of Evidence: Level IV, therapeutic case series	Rowe Walch Duplay recurrence mobility stability RTS (return to sport) pain X-ray CT scan	Reproducible, safe, with good cosmetic and functional outcomes	→
Mouchanta et al. [54]	73	3 wks	5 (7%)	Arthroscopic Latarjet procedure	A multicenter retrospective study	RTS, time to rugby practice, athletic level, patient satisfaction, recurrence, apprehension, SSV (subjective shoulder value), 3-month CT scan	Effective for rugby players; high RTP, low recurrence, high patient satisfaction	→
Meraner et al. [55]	132	0 wks	8 (6.1%)	Arthroscopic Latarjet procedure	A total of 132 shoulders retrospective study	The aim of this study is to evaluate the clinical outcomes and complications of the procedure, with a particular focus on the infection rate and nerve damage The DASH questionnaire was completed by 60% of the patients	Reliable for shoulder instability; prevents chronic luxation with low recurrence	→
Pelletier et al. [56]	40	3 wks	3 (7.5%)	Arthroscopic Cortical-Button Latarjet Procedure	This is a monocentric retrospective study including 40 patients	active range of motion apprehension test Rowe Walch-Duplay Subjective Shoulder Value Net Promoter Score. Radiologically, evolution of the bone graft and degenerative arthritis of the shoulder	95% RTP, 7.5% recurrence, 16% apprehension, 19% GH osteoarthritis, high satisfaction	↑
Tadeu et al. [57]	26	2 wks	0 (0%)	Arthroscopic Latarjet procedure with endobuttons	Methods: A retrospective study of 26 patients	DASH UCLA Rowe Visual Analog Scale (VAS) Short-Form 36 (SF36) Correct position and consolidation of the graft were evaluated	Effective, safe, good functional outcomes, enables early rehabilitation	→
Shao et al. [58]	425	?¿	1,50%	Arthroscopic Bristow versus Latarjet with screws or buttons	A prospective longitudinal	Recurrent dislocation, subluxation, and infections led to reoperations. Complications: 27.1% in Bristow, 25.6% in Latarjet, mainly graft-related (11.7%) and neurological (10.7%)	Suture-button Bristow has fewer complications than screw fixation	↓
Brzoska et at. [59]	46	4 wks	4 (8.7%)	Arthroscopic Latarjet procedure	Study Design: Case series; Level of evidence, 4. 50 months	Sport activity assessed via KJOC, RTS score, Constant-Murley, Walch-Duplay, ROM, complications, recurrence, and revisions	95.7% RTS after arthroscopic Latarjet, with occasional complications	→
Shao et al. [60]	30	4–6 wks	0 (0%)	Arthroscopic Latarjet procedure with modified button fixation	Retrospective study	UCLA ASES Rowe Radiologic assessment on 3D CT scan was performed preoperatively and postoperatively. Compli cations were also recorded	Modified suture-button Latarjet ensures stable fixation, good outcomes, low complications, and bone remodeling	↓
Zeng et al. [61]	37	4 wks	0 (0%)	Arthroscopic Latarjet procedure	Retrospective study	Walch-Duplay, SSV, Rowe, AROM, and 3D CT assessed graft position and bone resorption	Arthroscopic Latarjet + capsular repair shows good short-term outcomes; long-term effects need further study	→

↑: positive effect; →: no effect; ↓: negative effect; N: sample; IMT: immobilization time; EI: episodes of instability, including recurrent dislocations and subluxations. STechnique: surgical technique; SD: study design; V: variables; MR: Main results; EF: effect

### Risk of bias

**Fig. 3:**
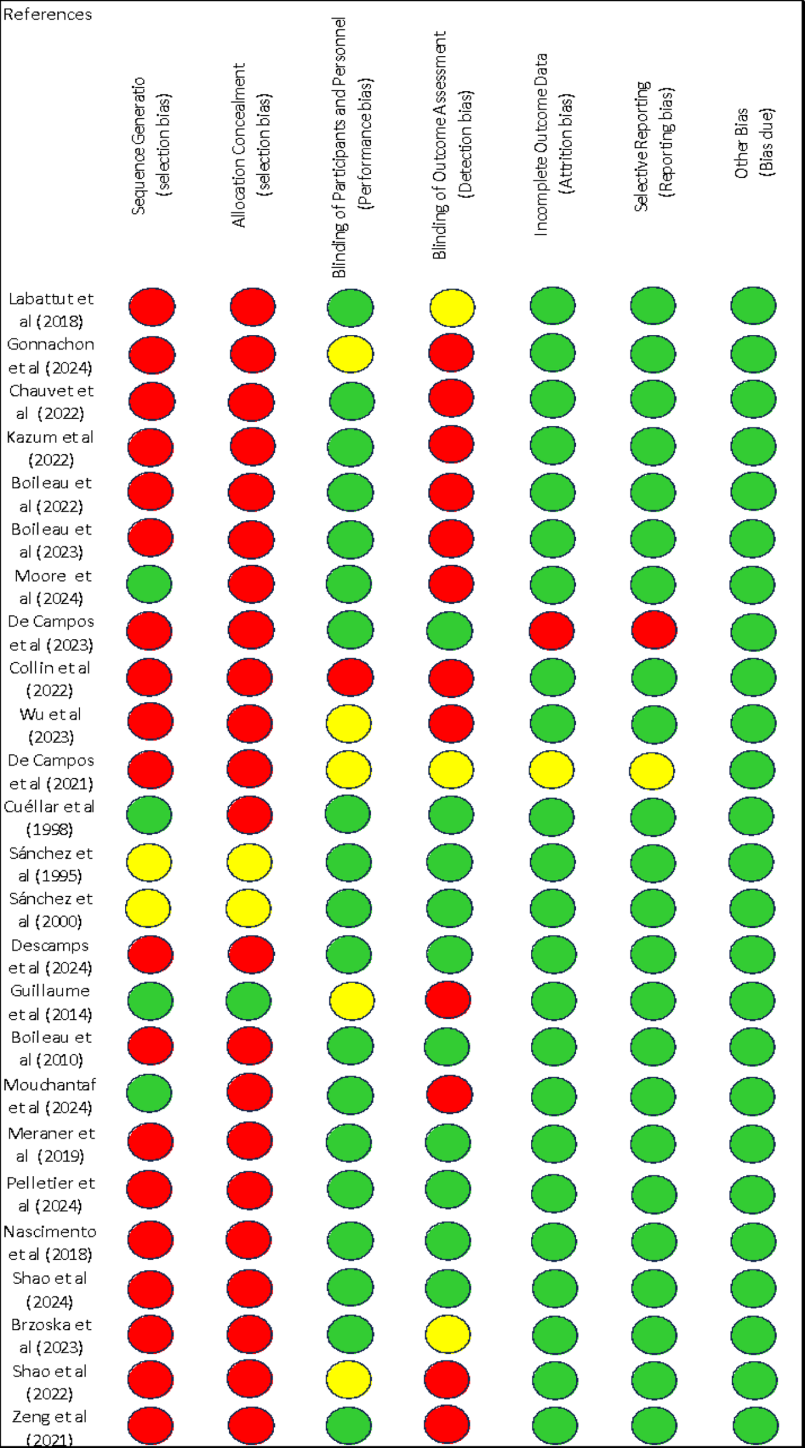
Risk of bias summary: authors’ judgments about each risk of bias item, presented as percentages across all included studies

**Fig. 4:**
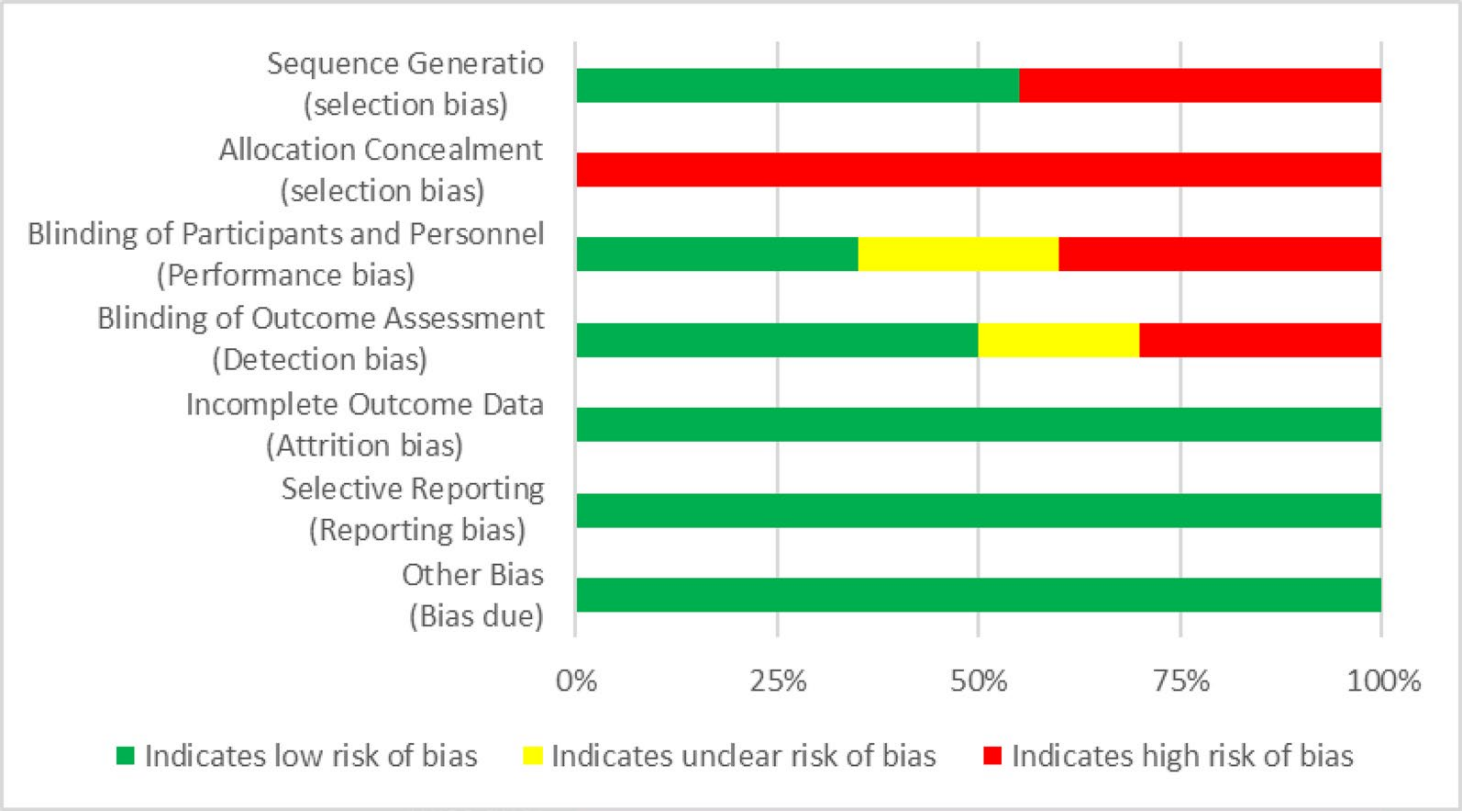
Risk of bias summary: authors’ judgments about each risk of bias item for each included study

The methodological quality and the risk of bias were evaluated following the guidelines of the Cochrane Collaboration [34]. For each investigation, the criteria were rated as “low” if they were met for a low risk of bias (unlikely to severely alter the results), or “high” if they indicated a high risk of bias (significantly weakening the reliability of the results). If the risk of bias was unknown, it was considered “not clear” (indicating uncertainty about the results). Every included study was assessed for the risk of bias [34]. The full assessments of study quality are shown in Figs. 3 and 4.

### Methodological quality assessment

The methodological quality of the included and analyzed studies ranged from 3 to 9 points, with an average of 6.92 points. The distribution was as follows: 1 study scored 3 points, 4 studies scored 5 points, 2 studies scored 6 points, 7 studies scored 7 points, 10 studies scored 8 points, and 1 study scored 9 points.

Despite some variation in item scores, there was notable consistency in certain criteria that were clearly met across studies. Specifically, item 4 (“the groups were similar at baseline in relation to the indicators of prognosis”), item 10 (“the study provides specific and variability measures for at least one key result”), and item 11 (“the results of statistical comparisons among groups were reported for at least one key result”) were consistently fulfilled. Conversely, none of the studies met item 3 (“the allocations were undisclosed”). Finally, one study met all quality criteria except for item 3 (Table 3).

**Table 3:** Results according to PEDro scale (*n* = 25)

Clinical trial	1	2	3	4	5	6	7	8	9	10	11	Total
Labattut et al. [37]	Yes	No	No	Yes	Yes	Yes	No	Yes	Yes	Yes	Yes	7
Gonnachon et al. [38]	Yes	No	No	Yes	No	No	No	Yes	Yes	Yes	Yes	5
Chauvet et al. [39]	Yes	No	No	Yes	Yes	Yes	No	Yes	Yes	Yes	Yes	7
Kazum et al. [40]	Yes	No	No	Yes	Yes	Yes	No	Yes	Yes	Yes	Yes	7
Boileau et al. [41]	Yes	No	No	Yes	Yes	Yes	No	Yes	Yes	Yes	Yes	7
Boileau et al. [42]	Yes	No	No	Yes	Yes	Yes	No	Yes	Yes	Yes	Yes	7
Moore et al. [43]	Yes	Yes	No	Yes	Yes	Yes	No	Yes	Yes	Yes	Yes	8
De Campos et al. [44]	Yes	No	No	Yes	Yes	Yes	Yes	No	No	Yes	Yes	6
Collin et al. [45]	Yes	No	No	Yes	No	No	No	Yes	Yes	Yes	Yes	5
Wu et al. [46]	Yes	No	No	Yes	No	No	No	Yes	Yes	Yes	Yes	5
De Campos et al. [47]	Yes	No	No	Yes	No	No	No	No	No	Yes	Yes	3
Cuellar et al. [48]	Yes	Yes	No	Yes	Yes	Yes	Yes	Yes	Yes	Yes	Yes	9
Sanchez et al. [49]	Yes	No	No	Yes	Yes	Yes	Yes	Yes	Yes	Yes	Yes	8
Sanchez et al. [50]	Yes	No	No	Yes	Yes	Yes	Yes	Yes	Yes	Yes	Yes	8
Descamps et al. [51]	Yes	No	No	Yes	Yes	Yes	Yes	Yes	Yes	Yes	Yes	8
Guillaume et al. [52]	Yes	Yes	No	Yes	No	No	No	Yes	Yes	Yes	Yes	6
Boileau et al. [53]	Yes	No	No	Yes	Yes	Yes	Yes	Yes	Yes	Yes	Yes	8
Mouchant et al. [54]	Yes	Yes	No	Yes	Yes	Yes	No	Yes	Yes	Yes	Yes	8
Meraner et al. [55]	Yes	No	No	Yes	Yes	Yes	Yes	Yes	Yes	Yes	Yes	8
Pelletier et al. [56]	Yes	No	No	Yes	Yes	Yes	Yes	Yes	Yes	Yes	Yes	8
Nascimento et al. [57]	Yes	No	No	Yes	Yes	Yes	Yes	Yes	Yes	Yes	Yes	8
Shao et al. [58]	Yes	No	No	Yes	Yes	Yes	Yes	Yes	Yes	Yes	Yes	8
Brzoska et al. [59]	Yes	No	No	Yes	Yes	Yes	No	Yes	Yes	Yes	Yes	7
Shao et al. [60]	Yes	No	No	Yes	No	No	No	Yes	Yes	Yes	Yes	5
Zeng et al. [61]	Yes	No	No	Yes	Yes	Yes	No	Yes	Yes	Yes	Yes	7

Yes: it presents the studied criterium. No: it does not present the studied criterium

1. The criteria of election were specified; 2. The subjects were randomly assigned to the groups; 3. The assignment was hidden; 4. The groups were similar at the beginning in relation to the most important indicators of prognosis; 5. All participants were blinded; 6. All the sports scientists providing therapy were blinded; 7. All assessors evaluating at least one of key results were blinded; 8. All the measures of at least one of the key results were obtained from more than 85% of the participants initially assigned to the groups; 9. The results of all the subjects receiving treatment or assigned to the control group were given, or when not possible, the data for at least one key result were analysed “in order to treat”; 10. The results of statistic comparisons among groups were reported for at least one key result; 11. The study provides specific and variability measures for at least one key result

**Fig. 5:**
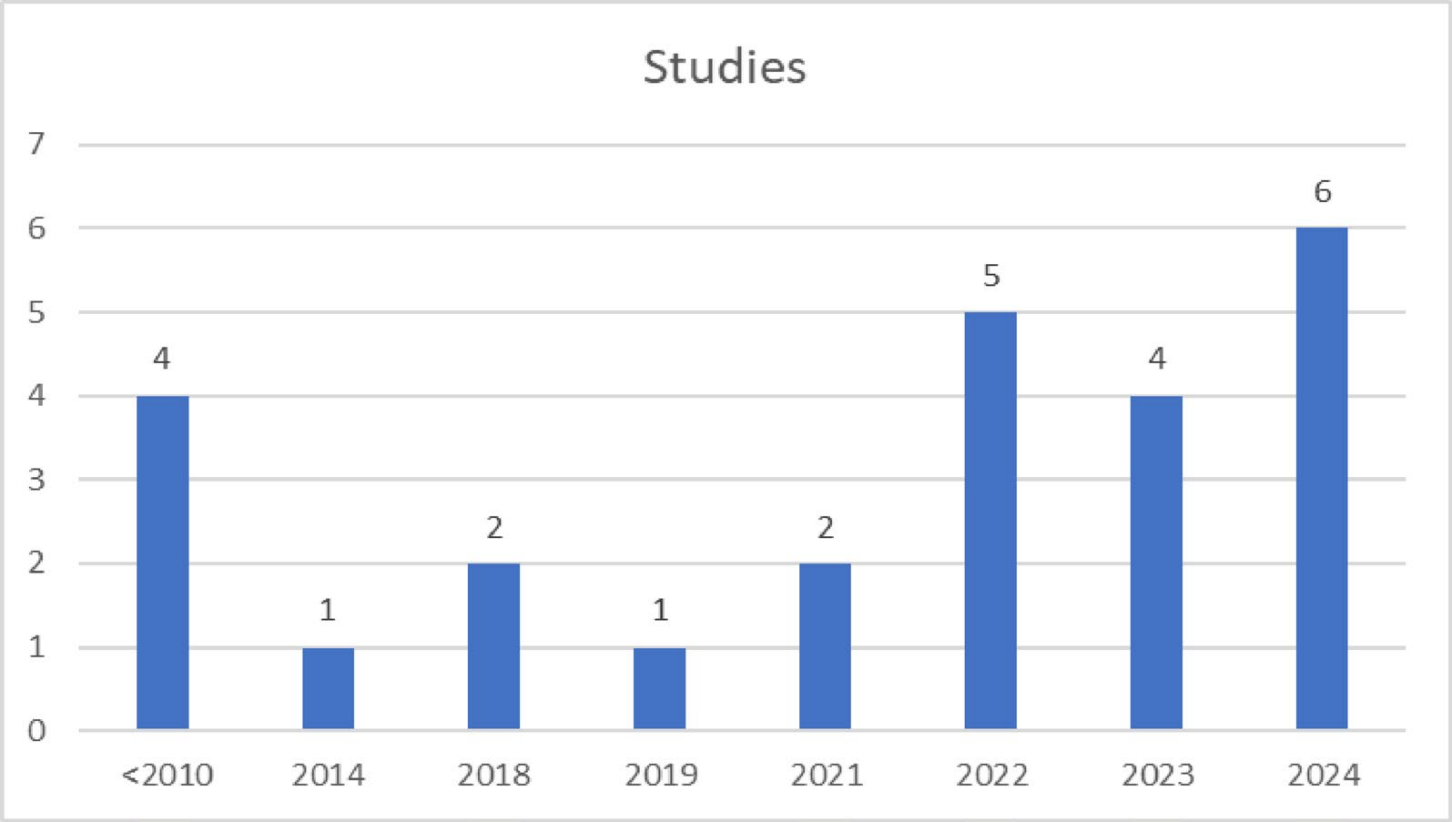
Chronology of the number of studies published on surgical techniques for treating shoulder instability

With regard to the chronology of the 25 articles studied (Fig. 5), 13 have been published in the last 17 years; 6 in 2024 [38, 43, 52, 55, 57, 59]; 4 in 2023 [42, 44, 46, 60]; 5 in 2022 [39–41, 45, 61]; and 2 in 2021 [47, 62]. The remaining research corresponds to 8 studies, which are divided into 1 in 2019 [56]; 2 in 2018 [37, 59]; 1 in 2014 [53]; and finally, 4 prior to 2010 [48, 50, 51, 54]. The above shows the great interest and importance of use of surgical techniques for the treatment of shoulder instability.

The reviewed studies highlight the clinical utility of these arthroscopic techniques, each offering specific benefits based on patient anatomy and functional needs. The overall evidence supports the effectiveness and safety of these ones. Each technique offers distinct advantages based on anatomical deficits and functional demands, emphasizing the need for personalized surgical planning in the management of anterior shoulder instability.

All the above information is summarized in the following Table 4:

**Table 4:** Summary of surgical techniques for treating shoulder instability

Nº Studies	Thematic	Conclusion
4	Dynamic Anterior Stabilization (DAS)	This technique would be indicated mainly in patients with recurrent shoulder instability with limited bone defects, associated with SLAP
11	Arthroscopic Latarjet	The technique is especially relevant when unipolar bone loss exceeds 20% of the glenoid surface, notably when associated with humeral bone loss in the form of an off-track Hill-Sachs lesion.
3	Arthroscopic Ligamentoplasty	Its primary indication would be in patients with high functional demand and multi-recurrent instability, particularly in those with poor or suboptimal capsuloligamentous tissue quality.
7	Arthroscopic Trillat	Particularly suited when the goal is to achieve a dynamic, “tenodesis-like” sling effect on the subscapularis, generated by medialization and distalization of the coracoid with the conjoint tendon, while preserving the native anatomy of the subscapularis and other soft tissues.

## Discussion

### Summary of main findings

The main aim of this of this systematic review was to collect, synthesize, and integrate international research published across various scientific databases on surgical techniques such as shoulder ligamentoplasty, arthroscopic Latarjet, dynamic anterior stabilization, and arthroscopic Trillat for the treatment of shoulder instability.

Anterior shoulder instability is a common condition affecting both the general population and athletes, and represents one of the most frequent causes of functional limitation in the upper limb. Its pathophysiology is based on an imbalance between the dynamic and static stabilizers of the glenohumeral joint, predisposing individuals to recurrent episodes of dislocation and subluxation. This condition, in addition to causing pain and disability, increases the risk of progressive joint deterioration and the development of glenohumeral arthropathy, reinforcing the need for effective and personalized therapeutic intervention [4, 62].

The surgical treatment of anterior instability has evolved significantly over recent decades, transitioning from highly invasive open procedures to advanced arthroscopic techniques aimed at maximizing joint stability while minimizing tissue damage [62]. Among the most widely used and studied techniques are DAS, arthroscopic Latarjet, arthroscopic ligamentoplasty, and arthroscopic Trillat. These techniques have proven effective in different patient subgroups depending on the specific characteristics of their instability, the presence of bone deficits, and the quality of the capsuloligamentous tissue [22–25].

These four techniques share the ability to achieve satisfactory outcomes in patients whose instability is influenced by both osseous and soft tissue risk factors. However, a key difference lies in the fact that ligamentoplasty is the only technique that does not alter the patient’s anatomy. It involves the repair of all possible structures, the potential execution of a remplissage to address humeral bone defects, and the addition of a ligamentous graft that does not modify the anatomy but acts as an adjunct to the patient’s natural structures. This also prevents glenohumeral dislocation in cases where abduction and external rotation are extreme enough for the humeral head to dislocate anteroinferiorly on the scapular glenoid [22].

This anatomical preservation is not present in DAS, Trillat, or arthroscopic Latarjet, establishing a significant distinction among these techniques. In the latter three, the anatomy is altered to enhance stabilization, and although the objective is effectively achieved, this factor is not without risks or potential future complications [22–25]. These must be carefully considered when determining the best surgical approach for each patient, particularly given that the target population is composed of young and active individuals.

The indicated measures were verified in terms of efficiency in the different studies analyzed in this review. There currently exist many literary proposals which attempt to consolidate these techniques in terms of prevention protocols, studying their effects in a complex manner. In spite of this, it was observed that preventive actions are not currently implemented systematically.

**Fig. 6:**
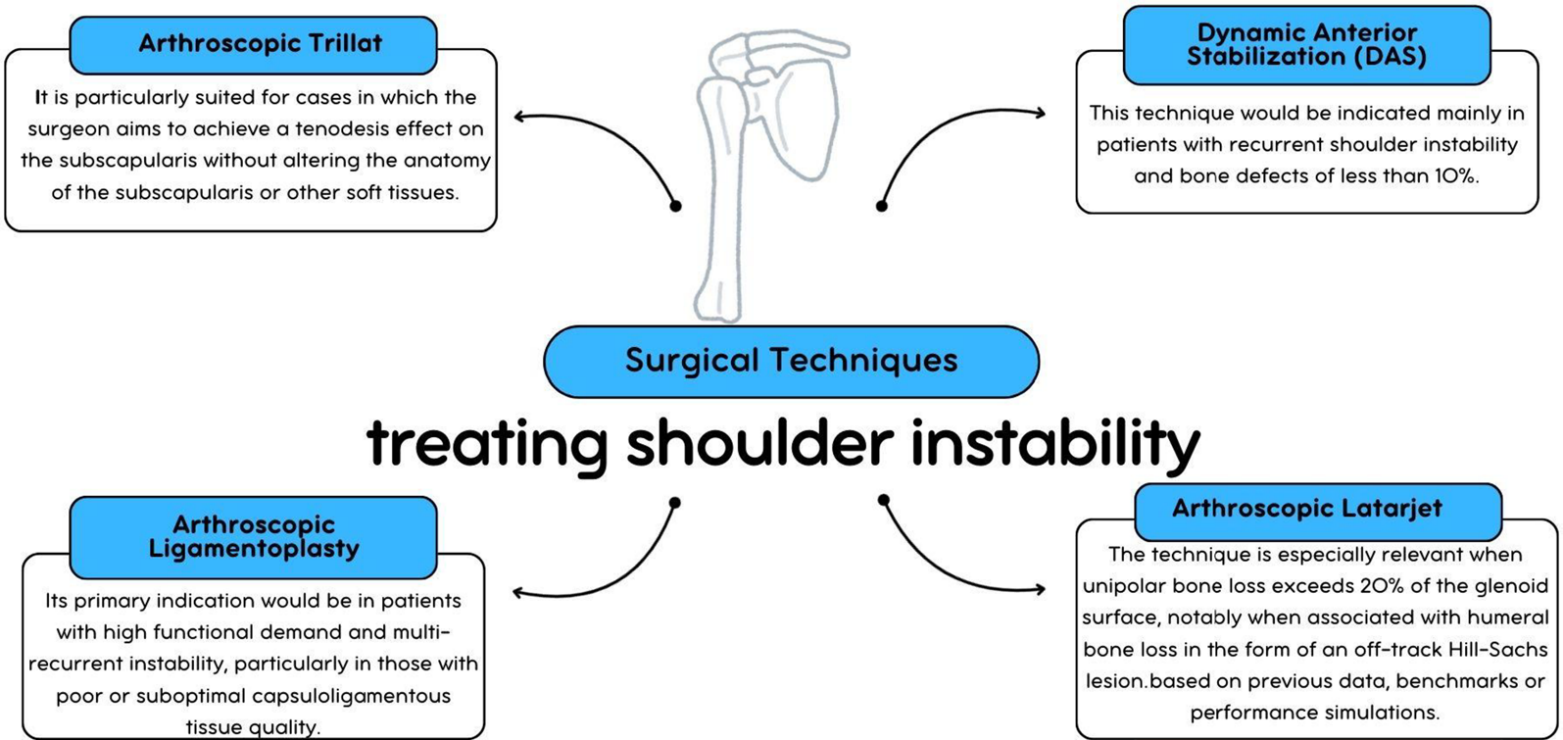
Surgical Techniques

Therefore, the current scientific literature describes different surgical approaches that aim to balance anatomical preservation, joint stability, and functional recovery (Fig. 6). However, there is a clear need for more robust comparative data, particularly through randomized controlled trials, to guide optimal technique selection. This systematic review seeks to address this gap by providing a comprehensive analysis of the available evidence, helping clinicians better understand the indications, outcomes, and limitations of each arthroscopic technique used in the management of anterior shoulder instability.

### Dynamic anterior stabilization (DAS)

The DAS technique has recently emerged as a less invasive alternative for shoulder stabilization in patients with anterior instability and subcritical bone loss. Its biomechanical principle involves transferring the long head of the biceps tendon through the subscapularis to create a sling effect that enhances joint stability under anterior stress. Unlike other techniques focused on osseous or capsular reconstruction, DAS utilizes the biceps’ dynamic stabilizing function to provide active control of humeral translation [23].

Recent studies [23, 46, 47] have shown promising results with this technique. Clara de Campos Azevedo et al. [47] reported a significant reduction in recurrence rates and considerable improvement in postoperative functional scores in patients with subcritical instability treated with DAS. Similarly, Collin et al. [23] found that this technique preserves the range of motion without compromising shoulder function, offering a key advantage over more invasive procedures. Wu et al. [46] observed comparable return-to-sport rates between DAS and other traditional techniques, highlighting its effectiveness in postoperative rehabilitation.

However, DAS still presents certain challenges and limitations. Its indication is restricted to patients with mild to moderate bone loss, as its stabilizing effect may be insufficient in cases of critical bone loss. Additionally, the long-term clinical outcomes remain uncertain and under evaluation, requiring further data collection to determine its efficacy compared to more established techniques [44].

This technique would be indicated mainly in patients with recurrent shoulder instability and bone defects of less than 10%, particularly when associated with pathology of the bicep’s tendon or its superior glenoid insertion, and when the goal is to address both conditions while adding a tenodesis effect or sling effect to the subscapularis tendon.

### Arthroscopic latarjet

Arthroscopic Latarjet is one of the most widely used techniques for treating anterior instability with critical bone loss. It involves the transfer of the coracoid process along with the conjoint tendon to the anteroinferior region of the glenoid, providing a dual stabilizing effect: the bone block increases contact surface area and prevents excessive humeral translation, while the tension generated by the conjoint tendon acts as a dynamic stabilizing mechanism [22]. This sling effect is the dynamic restraint created by the conjoint tendon as it courses inferior to the subscapularis after coracoid transfer. In abduction–external rotation, the tendon becomes tensioned and acts as an anterior buttress, compressing the humeral head against the glenoid and resisting anterior translation. This position-dependent mechanism complements the static bone effect of the coracoid block, enhancing stability in the at-risk arc of motion. It works synergistically with capsulolabral repair to increase concavity-compression and reduce the tendency for humeral head engagement. In short, it provides dynamic stabilization without relying solely on capsular tightening [21].

Recent studies [50, 51, 53] have confirmed the effectiveness of arthroscopic Latarjet in preventing recurrence. Descamps et al. [51] reported that suture fixation instead of screws significantly reduced postoperative complications related to graft migration, osteolysis, and consolidation defects, improving procedural safety. Dumont et al. [52] demonstrated that the recurrence rate following surgery is low and comparable to that of open Latarjet, with the additional advantage of reduced surgical aggression and shorter recovery time. Boileau et al. [53] highlighted that this procedure is not only safe and reproducible but also offers superior aesthetic and functional outcomes compared to open techniques.

Despite these benefits, arthroscopic Latarjet remains technically demanding. It requires a high level of surgical expertise. Additionally, patient selection must be meticulous, as graft resorption, hardware-related complications, and glenohumeral arthropathy may present long-term challenges [54, 63, 64].

This technique, although effective, is not without complications. These may include potential neurological sequelae, especially in patients undergoing revision surgery or presenting with recurrent instability following prior treatments. It is particularly indicated in cases of severe, recurrent instability with extensive capsular and labral damage. The technique is especially relevant when unipolar bone loss exceeds 20% of the glenoid surface, notably when associated with humeral bone loss in the form of an off-track Hill-Sachs lesion. It is a technique that alters the native anatomy and, although highly effective, offers few clear alternative options in the event of failure. This is particularly critical when the joint is already compromised by both the initial instability and the consequences of the procedure or its failure. Therefore, except in selected cases, it should be considered a second-line treatment option.

### Arthroscopic ligamentoplasty

Arthroscopic ligamentoplasty represents an intermediate surgical option between conventional capsulolabral repair and bone reconstruction techniques in the management of anterior shoulder instability. This technique aims to enhance capsular stabilization through the fixation of a synthetic or biological ligament graft. It increases resistance to humeral head translation while preserving joint mobility. It is the only one of the four techniques that does not alter the patient’s native anatomy. The procedure maintains bony integrity, reconstructs the capsuloligamentous structures, and adds mechanical support through the graft itself [23, 65].

Clinical studies [23, 50, 66] have demonstrated the potential benefits of arthroscopic ligamentoplasty. Cuéllar Gutiérrez et al. [48] reported favorable clinical outcomes in patients with recurrent anterior instability without significant bone loss, achieving a 90% satisfaction rate. These findings were supported by Sánchez et al., who described effective functional recovery and minimal postoperative morbidity in patients treated with synthetic anterior capsular reinforcement [23, 50]. Collectively, these results suggest that arthroscopic ligamentoplasty may be a safe and effective surgical option in selected patients. It expands the range of techniques available for shoulder stabilization.

However, the role of arthroscopic ligamentoplasty remains a subject of ongoing discussion within the orthopedic community. One of the primary concerns is the variability in graft integration, which may influence the long-term success and mechanical reliability of the procedure. While short- and mid-term results appear promising, further studies are warranted to assess long-term durability, outcomes, and complications, particularly regarding graft behavior over time [67].

Arthroscopic ligamentoplasty offers a distinct approach to shoulder stabilization by reinforcing the capsular structures without altering the native anatomy. Clinical data support its ability to achieve satisfactory functional recovery and high patient satisfaction in properly selected cases [48, 50]. Nevertheless, the long-term performance of the technique and the biological behavior of the graft require continued investigation to more clearly define its role in the surgical management of shoulder instability.

This technique should be more widely adopted because it is arthroscopic, it allows use of the subscapularis sling effect without altering its anatomy, is compatible with the arthroscopic performance of other techniques such as Remplissage, Bankart repair, and treatment of SLAP lesions, and does not modify the joint’s bony anatomy or the anatomy of any of its tendons [65].

Its primary indication would be in patients with high functional demand and multi-recurrent instability, particularly in those with poor or suboptimal capsuloligamentous tissue quality [23, 48, 50, 65, 66], considering tissue to be of suboptimal quality when, on prior imaging studies or during arthroscopic assessment, it shows severe fibrosis or disruption related to previous surgery; when it is insufficient to recreate the “bumper” effect; when it cannot be reduced to its anatomic footprint to permit reattachment and achieve appropriate tension of the glenohumeral ligaments; or when it is simply absent due to physical destruction from a high number of dislocations or from aggressive reductions. Congenital hypoplastic labrum and congenital absence of the labrum are also deemed suboptimal tissue quality.

It is also indicated in cases of subcritical glenoid bone loss of less than 20% [65]. This technique enables soft tissue repair where feasible and adds both a tenodesis and sling effect to the subscapularis without altering the patient’s native anatomy, as the ligament can be placed either through the subscapularis tendon or above its superior border. The implants used have minimal impact on bone stock. The procedure reinforces the anterior capsule and ligaments by introducing a controlled block to extreme external rotation and abduction. All of this is achieved through a fully arthroscopic approach [65]. Alternatively, it may be performed with a minimal axillary incision when the mini-invasive humeral fixation technique is chosen. Moreover, if the surgeon prefers to avoid the use of synthetic grafts due to concerns regarding their healing potential, the technique allows for the use of either an allograft or a tendon autograft. Therefore, it represents our treatment of choice in cases of severe instability in active patients with subcritical bone loss and compromised soft tissue quality [23, 65].

Finally, this technique can reinforce the anterior joint capsule in patients with multidirectional instability, a group with many treatment options but limited evidence to guide selection, and with heterogeneous, poorly standardized indications and outcomes [68].

### Arthroscopic trillat

The arthroscopic Trillat technique is a minimally invasive and promising surgical option for the treatment of anterior shoulder instability in selected patients without significant bone loss. This procedure involves an arthroscopic inferior closed-wedge osteoclasty (partial osteotomy) of the coracoid base to medialize and distalize the coracoid, thereby reducing the subcoracoid space and increasing tension on the conjoint tendon. By changing the coracoid position, a dynamic subscapularis–conjoint tendon sling is created (“sling effect”), which limits anterior humeral head translation and enhances joint stability [37]. The osteoclasis is secured by fixing the coracoid to the anterior scapular/glenoid neck with a coracoscapular screw or nail and in all-arthroscopic variants, with cannulated screws or low-profile suture-button constructs.

Clinical studies [39–41] have reported positive outcomes, particularly in patients with hyperlaxity or chronic post-traumatic anterior instability. Labattut et al. [37] demonstrated satisfactory short- and mid-term outcomes with a low complication profile, minimal surgical time, and early return to activity. Chauvet et al. [38] confirmed these results with a two-year follow-up, showing sustained shoulder stability and functional recovery. Boileau et al. [42] further highlighted the effectiveness of the technique in two distinct populations: young athletes with hyperlaxity and older patients with recurrent dislocations and massive irreparable rotator cuff tears (MIRCTs) [41]. Boileau et al. [42] highlighted the effectiveness of the Trillat procedure in two distinct groups: young hyperlax athletes and older patients with recurrent dislocations and massive irreparable rotator cuff tears (MIRCTs). In the latter scenario—anterior instability secondary to massive irreparable posterosuperior cuff tears with preserved motion—the humeral head slides over the anterior glenoid rim without producing a Bankart lesion, so a labral repair is not indicated; remplissage is also not feasible due to the pronounced posteromedial retraction of the infraspinatus. Because stability and function rely on a healthy subscapularis, procedures such as Latarjet risk compromising it and may precipitate a pseudoparalytic shoulder, whereas reverse shoulder arthroplasty is unwarranted when mobility is functional and painless. Accordingly, in our opinion, the Trillat procedure remains the main non-prosthetic option to treat recurrent instability in cuff-deficient shoulders.

Other indications for this technique include recurrent anterior instability in the absence of significant glenoid or humeral bone defects, especially in patients with hyperlaxity. Arthroscopy provides superior visualization of the glenohumeral joint, allowing accurate coracoid osteotomy and secure fixation. The outcomes are encouraging, with improved shoulder stability and low recurrence rates. Kazum et al. [40] found the procedure effective for anterior/inferior hyperlaxity, with minimal postoperative complications. Moore et al. [69] also reported high functional scores and rapid return to sports in a multicenter study involving athletic populations.

Regarding complications, the technique is generally safe. Gonnachon et al. [39] reported mild subscapularis atrophy in some patients at 6-month follow-up, but without associated strength deficits at 1 year, likely related to implant positioning.

In summary, the arthroscopic Trillat technique represents a valuable alternative to procedures like Latarjet in cases of functional instability without significant bone loss, MIRCTs, and hyperlaxity. Its minimally invasive nature allows for faster recovery and reduced morbidity. However, optimal results depend on proper patient selection and technical precision. Further high-quality comparative studies and long-term follow-up are necessary to define its definitive role in shoulder stabilization algorithms.

### Strengths, limitations, and future lines of research

This systematic review integrates and compares four advanced arthroscopic stabilization procedures, arthroscopic Latarjet, arthroscopic Trillat, DAS, and shoulder ligamentoplasty, synthesizing contemporary evidence to support patient-specific selection across subcritical and critical bone-loss scenarios. Its principal limitations mirror those of the current literature: heterogeneity in patient cohorts and indications, variability in follow-up, and nonstandardized outcome measures, along with constraints of the search/eligibility strategy that may have led to inadvertent omissions and limit cross-study comparability. Future research should prioritize Level-1 evidence, including well-powered randomized controlled trials and direct head-to-head comparative designs in clearly stratified cohorts, together with large prospective studies with long-term follow-up and core, standardized outcome sets (e.g., patient-reported outcome measures, return-to-sport rates, and uniform definitions of failure). Prespecified subgroup analyses (age, sport demands, laxity, degree of bone loss, on-/off-track status) are needed to refine indications, while advanced imaging to assess graft healing/integration and formal cost-effectiveness evaluations will enhance clinical and policy relevance. Finally, developing multivariable predictive tools and decision aids, coupled with continued refinement of minimally invasive techniques, should help personalize care, reduce complications, and accelerate recovery.

## Practical applications

From a clinical perspective, this systematic review offers valuable guidance for individualized surgical decision-making in the management of anterior shoulder instability. Arthroscopic Latarjet remains the gold standard for patients with significant glenoid bone loss. In contrast, DAS and ligamentoplasty represent promising alternatives for cases of subcritical instability. Understanding the specific advantages and limitations of each technique is essential to improving outcomes and minimizing complications. For example, suture-button fixation in the Latarjet procedure appears to reduce graft-related complications. Ligamentoplasty, by preserving native anatomy, is a suitable option for patients with hyperlaxity or those at risk of future instability.

For athletes and highly active individuals, arthroscopic Trillat and DAS may offer a faster return to sport due to their minimally invasive nature and better preservation of joint biomechanics. Ligamentoplasty is increasingly recognized for providing stability without permanently altering joint structures. Since it requires only a short period of immobilization and the implanted ligament limits pathological mobility, it enables high-demand patients to return to their previous activity levels more quickly.

Postoperative rehabilitation protocols must be tailored to the surgical technique used in order to optimize functional recovery and reduce recurrence rates. Beyond guiding treatment decisions and rehabilitation planning, this review also identifies gaps in current evidence, supports patient-centered communication regarding surgical options, and contributes to the development of future clinical practice guidelines based on high-quality evidence.

## Conclusions

The results of this systematic review of different studies present the evidence for the surgical techniques of shoulder ligamentoplasty, arthroscopic Latarjet, DAS, and arthroscopic Trillat for the treatment of shoulder instability. Arthroscopic ligamentoplasty excels in preserving the patient’s native anatomy. This not only maintains joint integrity but also allows for the possibility of alternative techniques in case of failure. The arthroscopic Trillat technique offers a minimally invasive option for anterior instability without significant bone loss. Although it alters the anatomy, the modification is limited and achieves a tenodesis-like effect on the subscapularis. The DAS technique uses the biceps tendon to provide dynamic stabilization and aims to generate a sling effect over the subscapularis. Finally, the Latarjet procedure remains the gold standard for treating anterior glenoid bone loss greater than 20%. Each surgical technique for anterior shoulder instability has specific implications. The choice of treatment should be based on an individualized assessment that considers bone loss, capsuloligamentous quality, and the patient’s functional demands.

## Data Availability

No datasets were generated or analysed during the current study.

## References

[1] Boone JL, Arciero RA. Management of failed instability surgery: how to get it right the next time. Orthop Clin North Am. 2010;41(3):367–79. 20497812 10.1016/j.ocl.2010.02.009

[2] Robinson CM, Howes J, Murdoch H, Will E, Graham C. Functional outcome and risk of recurrent instability after primary traumatic anterior shoulder dislocation in young patients. J Bone Joint Surg Am. 2006;88(11):2326–36. 17079387 10.2106/JBJS.E.01327

[3] Meller R, Krettek C, Gösling T, Wähling K, Jagodzinski M, Zeichen J. Recurrent shoulder instability among athletes: changes in quality of life, sports activity, and muscle function following open repair. Knee Surg Sports Traumatol Arthrosc. 2007;15(3):295–304. 16816984 10.1007/s00167-006-0114-x

[4] Ladd LM, Crews M, Maertz NA. Glenohumeral joint instability. Clin Sports Med. 2021;40(4):585–99. 34509200 10.1016/j.csm.2021.05.001

[5] Olds M, Donaldson K, Ellis R, Kersten P. In children 18 years and under, what promotes recurrent shoulder instability after traumatic anterior shoulder dislocation? A systematic review and meta-analysis of risk factors. Br J Sports Med. 2016;50(18):1135–41. 10.1136/bjsports-2015-095149. 26701925 10.1136/bjsports-2015-095149

[6] Kurokawa D, Yamamoto N, Nagamoto H, et al. The prevalence of a large Hill-Sachs lesion that needs to be treated. J Shoulder Elb Surg. 2013;22(9):1285–9. 10.1016/j.jse.2012.12.033 23466174

[7] Arenas-Miquelez A, Barco R, Cabo FJ, Hachem A. Management of bone loss in anterior shoulder instability. Bone Joint J. 2024;106–B(10):1100–10. 10.1302/0301-620X.106B10.BJJ-2024-0501.R1. 39348897 10.1302/0301-620X.106B10.BJJ-2024-0501.R1

[8] Locher J, Wilken F, Beitzel K, Buchmann S, Wieser K. Hill-Sachs off-track lesions as risk factor for recurrence of instability after arthroscopic Bankart repair. Arthroscopy. 2016;32(8):1993–9. 10.1016/j.arthro.2016.02.024. 27161511 10.1016/j.arthro.2016.03.005

[9] Shaha JS, Cook JB, Song DJ, et al. Redefining critical bone loss in shoulder instability: functional outcomes worsen with subcritical bone loss. Am J Sports Med. 2015;43(7):1719–25. 10.1177/0363546515578250. 25883168 10.1177/0363546515578250

[10] Dickens JF, Owens BD, Cameron KL, et al. The effect of subcritical bone loss and exposure on recurrent instability after arthroscopic Bankart repair in intercollegiate American football. Am J Sports Med. 2017;45(8):1769–75. 28474965 10.1177/0363546517704184

[11] Di Giacomo G, Itoi E, Burkhart SS. Evolving concept of bipolar bone loss and the Hill-Sachs lesion: from engaging/non-engaging lesion to on-track/off-track lesion. Arthroscopy. 2014;30(1):90 – 8. 10.1016/j.arthro.2013.10.004. PMID: 24384275. 10.1016/j.arthro.2013.10.004 24384275

[12] Papalia R, Franceschi F, Diaz Balzani L, D’Adamio S, Denaro V, Maffulli N. The arthroscopic treatment of shoulder instability: bioabsorbable and standard metallic anchors produce equivalent clinical results. Arthroscopy. 2014;30(9):1173-83. 10.1016/j.arthro.2014.03.030. Epub 2014 Jun 3. PMID: 24933591. 10.1016/j.arthro.2014.03.030 24933591

[13] Haskel JD, Colasanti CA, Hurley ET, Matache BA, Jazrawi LM, Meislin RJ. Arthroscopic latarjet procedure: indications, techniques, and outcomes. JBJS Rev. 2021;9(3):e2000123. 10.2106/JBJS.RVW.20.00123. 10.2106/JBJS.RVW.20.00071 33690241

[14] Arciero RA, Parrino A, Bernhardson AS, et al. The effect of a combined glenoid and Hill-Sachs defect on glenohumeral stability: a Biomechanical cadaveric study using 3-dimensional modeling of 142 patients. Am J Sports Med. 2015;43(6):1422–9. Lafosse Harris JDdoi:10.1177/0363546515574677. 25794869 10.1177/0363546515574677

[15] Gouveia K, Abidi SK, Shamshoon S, et al. Arthroscopic Bankart repair with remplissage in comparison to bone block augmentation for anterior shoulder instability with bipolar bone loss: a systematic review. Arthroscopy. 2021;37(2):706–17. 10.1016/j.arthro.2020.08.033. 32911004 10.1016/j.arthro.2020.08.033

[16] Di Giacomo G, Pugliese M, Lie DTT, et al. How to handle minor and major bone loss d through the lens of arthroscopy. EFORT Open Rev. 2024;9(9):923–32. 10.1530/EOR-23-0208. 39222335 10.1530/EOR-23-0208 PMC11457812

[17] Hohmann E, Tetsworth K, Glatt V. Open versus arthroscopic surgical treatment for anterior shoulder dislocation: a comparative systematic review and meta-analysis over the past 20 years. J Shoulder Elb Surg. 2017;26(10):1873–80. 10.1016/j.jse.2017.04.009. 10.1016/j.jse.2017.04.009 28688936

[18] Harris JD, Gupta AK, Mall NA, Abrams GD, McCormick FM, Cole BJ, et al. Long-term outcomes after Bankart shoulder stabilization. Arthroscopy. 2013;29(5):920–33. 23395467 10.1016/j.arthro.2012.11.010

[19] Zimmermann SM, Scheyerer MJ, Farshad M, Catanzaro S, Rahm S, Gerber C. Long-Term restoration of anterior shoulder stability: A retrospective analysis of arthroscopic Bankart repair versus open latarjet procedure. J Bone Joint Surg Am. 2016;98(23):1954–61. 10.2106/JBJS.15.01398. 27926676 10.2106/JBJS.15.01398

[20] Wang L, Liu Y, Su X, Liu S. A Meta-Analysis of arthroscopic versus open repair for treatment of Bankart lesions in the shoulder. Med Sci Monit. 2015;21:3028–35. 10.12659/MSM.894346. PMID: 26446430; PMCID: PMC4603609. 26446430 10.12659/MSM.894346 PMC4603609

[21] Rollick NC, Ono Y, Kurji HM, Nelson AA, Boorman RS, Thornton GM, Lo IK. Long-term outcomes of the Bankart and latarjet repairs: a systematic review. Open Access J Sports Med. 2017;8:97–105. PMID: 28450792; PMCID: PMC5399974. 28450792 10.2147/OAJSM.S106983 PMC5399974

[22] Lafosse L, Boyle S, Gutierrez-Aramberri M, Shah A, Meller R. Arthroscopic latarjet procedure. Orthop Clin North Am. 2010;41(3):393–405. 10.1016/j.ocl.2010.02.001. 20497814 10.1016/j.ocl.2010.02.004

[23] Sánchez AM. Luxación recidivante de hombro. Cirugía artroscópica Con Refuerzo capsular anterior sintético. Cuad Artrosc. 1995;2(2):46–52.

[24] Swan J, et al. Arthroscopic trillat procedure: a guided technique. Arthrosc Tech. 2020;9(4):e513–9. 32368472 10.1016/j.eats.2019.12.004 PMC7189268

[25] Shekhbihi A, Bauer S, Walch A, Reichert W, Walch G, Boileau P. The trillat procedure: the man and the technique revisitetematic reviews of interventions. Version 5.1.0. London: The Cochrane Collaboration; 2011. pp. 1–639. 10.1530/EOR-23-0208.

[26] Collin P, Lädermann A. Dynamic anterior stabilization using the long head of the biceps for anteroinferior glenohumeral instability. Arthrosc Tech. 2017;7(1):e39–44. 10.1016/j.eats.2017.08.049. 29552467 10.1016/j.eats.2017.08.049 PMC5852254

[27] Secci G, Schippers P, Biégun M, Mouchantaf M, Boileau P. The trillat procedure: a systematic review of complications and outcome. JSES Rev Rep Tech. 2024;4(4):694–702. 10.1016/j.xrrt.2024.06.011. PMID: 39474170; PMCID: PMC11514100. 39474170 10.1016/j.xrrt.2024.06.011 PMC11514100

[28] Jain N, McKeeman J, Higgins M, Johnson A, Smith T, Waterman B. Dynamic anterior shoulder stabilization using a long head of the biceps transfer and Bankart repair improves clinical outcomes in patients with subcritical bone loss: A systematic review. Arthrosc Sports Med Rehabil. 2025;7(3):101141. PMID: 40692939; PMCID: PMC12276567. 40692939 10.1016/j.asmr.2025.101141 PMC12276567

[29] Deng Z, Zheng Y, Su J, et al. Open versus arthroscopic latarjet for recurrent anterior shoulder instability: A systematic review and Meta-analysis. Orthop J Sports Med. 2023;11(5). 10.1177/23259671231174476. 10.1177/23259671231174476 PMC10280521 37346777

[30] Liberati A, Altman DG, Tetzlaff J, Mulrow C, Gøtzsche PC, Ioannidis JP, et al. The PRISMA statement for reporting systematic reviews and meta-analyses of studies that evaluate health care interventions: explanation and elaboration. Ann Intern Med. 2009;151(4):W65–94. 10.1136/bmj.b2700. 19622512 10.7326/0003-4819-151-4-200908180-00136

[31] Higgins JPT, Green S. Cochrane Handbook for Systematic Reviews of Interventions Version 5.1.0. Cochrane (2011). pp. 1–639.

[32] O’Connor D, Green S, Higgins JPT. Defining the review question and developing criteria for including studies. In: Higgins JPT, Green S, editors. Cochrane handbook for systematic reviews of interventions. Chichester: Wiley-Blackwell; 2008. pp. 81–94.

[33] Greenhalgh T, Peacock R. Effectiveness and efficiency of search methods in systematic reviews of complex evidence: audit of primary sources. BMJ. 2005;331(7524):1064–5. 16230312 10.1136/bmj.38636.593461.68 PMC1283190

[34] Higgins JP. Cochrane handbook for systematic reviews of interventions. Cochrane Collaboration and John Wiley & Sons Ltd; 2008.

[35] Verhagen AP, de Vet HC, de Bie RA, Kessels AG, Boers M, Bouter LM, Knipschild PG. The Delphi list: a criteria list for quality assessment of randomized clinical trials for conducting systematic reviews developed by Delphi consensus. J Clin Epidemiol. 1998;51(12):1235–41. 10.1016/S0895-4356(98)00131-0. 10086815 10.1016/s0895-4356(98)00131-0

[36] Ap V, Delphi T, Alt Murphy M, Resteghini C, Feys P, Lamers I. An overview of systematic reviews on upper extremity outcome measures after stroke. BMC Neurol. 2015;15:29. 10.1186/s12883-015-0292-6. 25880033 10.1186/s12883-015-0292-6 PMC4359448

[37] . Labattut L. Arthroscopy-assisted trillat procedure for anterior shoulder instability: surgical technique and preliminary clinical results. Orthop Traumatol Surg Res. 2018;104(6):811–6. 10.1016/j.otsr.2017.12.022. 29578105 10.1016/j.otsr.2017.12.022

[38] Gonnachon A, Michon B, Savoye-Laurens T, Colombi R, Baulot E, Labattut L, Martz P. Subscapularis atrophy and function after arthroscopic trillat procedure. Orthop Traumatol Surg Res. 2023;109(5):103961. 10.1016/j.otsr.2023.103961. 10.1016/j.otsr.2024.103961 39059546

[39] Chauvet T, Labattut L, Colombi R, Baudin F, Baulot E, Martz P. Arthroscopic trillat technique for chronic post-traumatic anterior shoulder instability: outcomes at 2 years of follow-up. J Shoulder Elb Surg. 2022;31(3):e270–8. 10.1016/j.jse.2021.12.007. 10.1016/j.jse.2021.12.007 35017078

[40] Kazum E, Martinez-Catalan N, Oussama R, Eichinger JK, Werthel JD, Valenti P. Arthroscopic trillat procedure combined with capsuloplasty: an effective treatment modality for shoulder instability associated with hyperlaxity. Knee Surg Sports Traumatol Arthrosc. 2022;30(6):2067–73. 10.1007/s00167-021-06752-z. 34655309 10.1007/s00167-021-06752-z

[41] Boileau P, Clowez G, Bouacida S, Walch G, Schwartz DG, Trojani C. The arthroscopic trillat procedure is a valuable and durable treatment option for recurrent anterior instability associated with massive irreparable cuff tears. Arthroscopy. 2023;39(4):935–45. 10.1016/j.arthro.2022.10.045. 36370919 10.1016/j.arthro.2022.10.045

[42] Boileau P, Clowez G, Bouacida S, Walch G, Trojani C, Schwartz DG. The arthroscopic trillat procedure is a valuable treatment option for recurrent anterior instability in young athletes with shoulder hyperlaxity. Arthroscopy. 2023;39(4):948–58. 10.1016/j.arthro.2022.10.046. 36368519 10.1016/j.arthro.2022.10.046

[43] Moore F, Labattut L, Chauvet T, Bordet A, Martz P. Arthroscopic trillat technique for chronic anterior shoulder instability: outcomes at 2-year follow-up in 74 at-risk sports patients. J Shoulder Elb Surg. 2025;34(5):1225–35. 10.1016/j.jse.2024.08.029. 10.1016/j.jse.2024.08.029 39396610

[44] de Campos Azevedo C, Ângelo AC. Onlay dynamic anterior stabilization with biceps transfer for the treatment of anterior glenohumeral instability produces good clinical outcomes and successful healing at a minimum 1 year of follow-up. Arthrosc Sports Med Rehabil. 2023;5(2):e445–57. 10.1016/j.asmr.2023.01.012. 37101880 10.1016/j.asmr.2023.01.012 PMC10123435

[45] Collin P, Nabergoj M, Denard PJ, Wang S, Bothorel H, Lädermann A. Arthroscopic biceps transfer to the glenoid with Bankart repair grants satisfactory 2-year results for recurrent anteroinferior glenohumeral instability in subcritical bone loss. Arthroscopy. 2022;38(6):1766–71. 10.1016/j.arthro.2021.11.043. 34883198 10.1016/j.arthro.2021.11.043

[46] Wu C, Xu J, Fang Z, Chen J, Ye Z, Wang L, et al. Arthroscopic dynamic anterior stabilization using either long head of the biceps or conjoined tendon transfer for anterior shoulder instability results in a similarly low recurrence rate. Arthroscopy. 2023;39(7):1618–27. 10.1016/j.arthro.2022.12.040. 36708745 10.1016/j.arthro.2022.12.040

[47] de Campos Azevedo C, Ângelo AC. All-suture anchor dynamic anterior stabilization produced successful healing of the biceps tendon: a report of 3 cases. JBJS Case Connect. 2021;11(1):e2000149. 10.2106/JBJS.CC.20.00149. 10.2106/JBJS.CC.20.00149 33599463

[48] Cuéllar Gutiérrez R, García Gutiérrez A, Silió Ochandiano F, Albillos Bartolomé FJ, Usabiaga Zarranz J. Refuerzo capsular anterior de dacrón En El Tratamiento de La luxación recidivante de hombro Tipo atraumático. Rev Esp Cir Ortop Traumatol. 1998;43(3):196–204.

[49] Sánchez M, Cuéllar R, García A, Albillos J. Anterior stabilization of the shoulder by means of an artificial capsular reinforcement and arthroscopy—Part I: surgical technique. J long Term Eff Med Implants. 2000;10(3):187–97.

[50] Sánchez M, Cuéllar R, Garcia A, Albillos J, Azofra J. Anterior stabilization of the shoulder by means of an artificial capsular reinforcement and Arthroscopy— part II: ^1^ clinical results. J long Term Eff Med Implants. 2000;10(3).

[51] Descamps J, Greco V, Chelli M, Boileau P. The arthroscopically guided Bristow-Latarjet procedure with cortical button fixation: a minimum 10-year follow-up. Am J Sports Med. 2024;52(11):2815–25. 10.1177/03635465241263590. 39221758 10.1177/03635465241263590

[52] Dumont GD, Fogerty S, Rosso C, Lafosse L. The arthroscopic latarjet procedure for anterior shoulder instability: 5-year minimum follow-up. Am J Sports Med. 2014;42(10):2560–6. 10.1177/0363546514544682. 25117728 10.1177/0363546514544682

[53] Boileau P, Mercier N, Roussanne Y, Thélu CE, Old J. Arthroscopic Bankart-Bristow-Latarjet procedure: the development and early results of a safe and reproducible technique. Arthroscopy. 2010;26(11):1434–50. 10.1016/j.arthro.2010.07.011. 21035007 10.1016/j.arthro.2010.07.011

[54] Mouchantaf M, Bastard C, Corsia S, Métais P, Nourissat G. High rates of return to play and low recurrence rate after arthroscopic latarjet procedure for anterior shoulder instability in rugby players. Arthrosc Sports Med Rehabil. 2024;6(2):100912. 10.1016/j.asmr.2024.100912. 38590787 10.1016/j.asmr.2024.100912 PMC10999816

[55] Meraner D, Smolen D, Sternberg C, Thallinger C, Hahne J, Leuzinger J. 10 years of arthroscopic latarjet procedure: outcome and complications. Indian J Orthop 2019 Jan-Feb;53(1):102–10. 10.4103/ortho.IJOrtho_273_17 10.4103/ortho.IJOrtho_273_17 PMC6394195 30905989

[56] Pelletier J, Barret H, Dalmas Y, Hamzaoui H, Mansat P, Bonnevialle N. Outcomes of arthroscopic cortical-button latarjet procedure with minimum 5-year follow-up. J Shoulder Elb Surg. 2024;33(–):1–9. 10.1016/j.jse.2024.08.041. 10.1016/j.jse.2024.08.041 39427731

[57] Nascimento AT, Claudio GK, Rocha PB, Zumárraga JP, Camargo OP. Arthroscopic latarjet technique combined with endobuttons: functional outcomes in 26 cases. Acta Ortop Bras. 2018;26(5):328–31. 10.1590/1413-785220182605208650. 30464715 10.1590/1413-785220182605208650 PMC6220660

[58] Shao Z, Zhao Y, Luo H, Jiang Y, Song Q, Cheng X, et al. Clinical and radiologic outcomes of all-arthroscopic latarjet procedure with modified suture button fixation: excellent bone healing with a low complication rate. Arthroscopy. 2022;38(7):2157–65. 10.1016/j.arthro.2022.01.020. 35093498 10.1016/j.arthro.2022.01.020

[59] Brzoska R, Laprus H, Malik SS, Solecki W, Juszczak B, Blasiak A. Return to preinjury-level sports after arthroscopic latarjet for recurrent anterior shoulder instability in professional athletes. Orthop J Sports Med. 2023;11(5):23259671231166371. 10.1177/23259671231166371. 37162759 10.1177/23259671231166371 PMC10164259

[60] Shao Z, Jiang Y, Song Q, Wang H, Luo H, Cheng X, et al. Short-term complications of arthroscopic Bristow or latarjet procedure with screw versus suture-button fixation: a prospective study of 308 consecutive cases by a single surgeon. J Bone Joint Surg Am. 2024;106(19):1776–84. 10.2106/JBJS.23.00390. 39325870 10.2106/JBJS.23.00390

[61] Zeng Z, Liu C, Liu Y, Huang Y. Early outcomes of the arthroscopic latarjet procedure in a series of 37 patients with shoulder instability. BMC Musculoskelet Disord. 2021;22:845. 10.1186/s12891-021-04726-3. 34600519 10.1186/s12891-021-04726-3 PMC8487569

[62] Hovelius L, Saeboe M. Neer award 2008: arthropathy after primary anterior shoulder dislocation—223 shoulders prospectively followed up for twenty-five years. J Shoulder Elb Surg. 2009;18(3):339–47. 10.1016/j.jse.2008.10.007. 10.1016/j.jse.2008.11.004 19254851

[63] Castricini R, Longo UG, Petrillo S, Candela V, De Benedetto M, Maffulli N, Denaro V. Arthroscopic latarjet for recurrent shoulder instability. Med (Kaunas). 2019;55(9):582. 10.3390/medicina55090582. PMID: 31514425; PMCID: PMC6781242. 10.3390/medicina55090582 PMC6781242 31514425

[64] Longo UG, Loppini M, Rizzello G, Ciuffreda M, Maffulli N, Denaro V, Latarjet. Bristow, and Eden-Hybinette procedures for anterior shoulder dislocation: systematic review and quantitative synthesis of the literature. Arthroscopy. 2014;30(9):1184 – 211. doi: 10.1016/j.arthro.2014.04.005. Epub 2014 Jun 4. PMID: 24907025. 10.1016/j.arthro.2014.04.005 24907025

[65] Gómez Cimiano FJ. Ligamentoplastia de hombro, 25 años de Una Joven técnica. Rev Esp Artrosc Cir Articul. 2018;25(Supl1):59–66. 10.24129/j.reaca.25e62.fs1802010.

[66] Achalandabaso J, Golanó P, Escobar E, Uribarri J, Fariñas O. Tratamiento quirúrgico de La inestabilidad anterior de hombro mediante Refuerzo capsular Con tendones de La Pata de Ganso. Cuad Artrosc. 2001;8(2):10–8.

[67] Batty LM, Norsworthy CJ, Lash NJ, Wasiak J, Richmond AK, Feller JA. Synthetic devices for reconstructive surgery of the cruciate ligaments: a systematic review. Arthroscopy. 2015;31(5):957–68. 10.1016/j.arthro.2014.11.022. 25620500 10.1016/j.arthro.2014.11.032

[68] Longo UG, Rizzello G, Loppini M, Locher J, Buchmann S, Maffulli N, Denaro V. Multidirectional instability of the shoulder: A systematic review. Arthroscopy. 2015;31(12):2431–43. 10.1016/j.arthro.2015.06.006. Epub 2015 Jul 21. PMID: 26208802. 26208802 10.1016/j.arthro.2015.06.006

[69] Moore F et al. Arthroscopic trillat technique: multicenter retrospective study. J Shoulder Elb Surg. 2024 [In press].

